# MTRSRP: Joint Design of Multi-Triangular Ring and Self-Routing Protocol for BLE Networks

**DOI:** 10.3390/s25154773

**Published:** 2025-08-03

**Authors:** Tzuen-Wuu Hsieh, Jian-Ping Lin, Chih-Min Yu, Meng-Lin Ku, Li-Chun Wang

**Affiliations:** 1 School of Big Data, Fuzhou University of International Studies and Trade, Fuzhou 350200, China; xiezunwu@fzfu.edu.cn; 2 College of Information Engineering, Yango University, Fuzhou 350015, China; jplin@ygu.edu.cn; 3 Department of Information and Computer Engineering, Chung Yuan Christian University, Taoyuan 320314, Taiwan; 4 Department of Communication Engineering, National Central University, Zhongli 320317, Taiwan; mlku@ce.ncu.edu.tw; 5 Department of Electrical and Computer Engineering, National Yang Ming Chiao Tung University, Hsinchu 300093, Taiwan; wang@nycu.edu.tw

**Keywords:** multi-triangular ring, decentralized strategy, scatternet formation, self-routing protocol

## Abstract

This paper presents the multi-triangular ring and self-routing protocol (MTRSRP), which is a new decentralized strategy designed to boost throughput and network efficiency in multiring scatternets. MTRSRP comprises two primary phases: leader election and scatternet formation, which collaborate to establish an effective multi-triangular ring topology. In the leader election phase, nodes exchange broadcast messages to gather neighbor information and elect coordinators through a competitive process. The scatternet formation phase determines the optimal number of rings based on the coordinator’s collected node information and predefined rules. The master nodes then send unicast connection requests to establish piconets within the scatternet, following a predefined role table. Intra- and inter-bridge nodes were activated to interconnect the piconets, creating a cohesive multi-triangular ring scatternet. Additionally, MTRSRP incorporates a self-routing addressing scheme within the triangular ring architecture, optimizing packet transmission paths and reducing overhead by utilizing master/slave relationships established during scatternet formation. Simulation results indicate that MTRSRP with dual-bridge connectivity outperforms the cluster-based on-demand routing protocol and Bluetooth low-energy mesh schemes in key network transmission performance metrics such as the transmission rate, packet delay, and delivery ratio. In summary, MTRSRP significantly enhances throughput, optimizes routing paths, and improves network efficiency in multi-ring scatternets through its multi-triangular ring topology and self-routing capabilities.

## 1. Introduction

The rapid evolution and widespread adoption of Internet of Things (IoT) technology have established Bluetooth low-energy (BLE) as a critical facilitator for connecting diverse smart devices. The distinct advantages of BLE include its low power consumption, cost-effectiveness, broad compatibility, and wide-ranging application potential, making it indispensable in domains such as smart homes, healthcare, and industrial control [1]. However, the continuous evolution of Bluetooth technology introduces new devices and standards, posing challenges to ensuring seamless compatibility between legacy and modern devices and effectively integrating and managing these devices. In addition, the growing complexity of application scenarios requires designing an optimal network configuration that entails identifying optimal communication protocols, streamlining data transmission pathways, and implementing effective device management within the network.

In BLE technology, piconets with different frequency-hopping channels can be connected using bridge nodes, thereby establishing a more extensive scatternet network with increased capacity. These bridge nodes are assigned distinct roles, such as a master/slave (M/S) bridge or slave/slave (S/S) bridge. An M/S bridge acts as a master node in one piconet and slave node in another, whereas an S/S bridge serves as a slave node across multiple piconets. A broader multi-hop subnet can be created by employing bridge nodes, thereby facilitating the implementation of mesh topology control and multi-hop routing functionalities in BLE devices. Research on multi-hop routing networks has primarily focused on topology construction and effective routing strategies. This involves efficiently organizing individual piconets, integrating them into a mesh network, and achieving efficient message transmission within a well-structured BLE network [2].

Various formation architectures in wireless networks can be categorized into tree [3], ring [4], and mesh [5] topologies. The tree structure is simple and suitable for creating a scatternet, whereas the ring topology offers straightforward routing but can result in longer transmission paths as the network expands. In contrast, mesh connections can reduce packet delays by shortening path lengths, but they also present complexity during formation. A novel approach integrates the tree, ring, and mesh structures into specialized formations to address this issue. For example, a hybrid ring tree (HRT) setup deploys a ring subnet in dense areas and extends tree subnets to cover sparser regions [6]. Another innovation is the dual-ring tree topology [7], which is suitable for distributed scatternet construction in single- or multi-hop communication scenarios. Furthermore, efforts are underway to integrate mesh and ring subnets and explore a hybrid mesh ring topology [8] to optimize mesh links within a single configuration.

Efficient routing within a scatternet is crucial and has led to the development of various routing protocols tailored for Bluetooth networks. These protocols include proactive [3,6], reactive [9,10], and hybrid schemes. Proactive protocols utilize local or global routing tables to create spanning tree scatternets, thereby facilitating packet forwarding. In contrast, reactive protocols dynamically discover routes that are effective in small-scale networks but may suffer from increased overhead as the network size increases. To mitigate this concern, a cluster-based flooding scheme reduces packet flooding during route discovery, thereby improving the routing performance. In addition, a self-routing protocol combined with a layer-based formation algorithm and preconfigured parameters minimizes route discovery overhead, enhancing network scalability and routing efficiency [11].

In this paper, we propose a multi-triangular ring and self-routing protocol (MTRSRP), which is a decentralized strategy designed to improve throughput and network efficiency in multi-ring scatternets. MTRSRP comprises two phases, namely, the leader election phase and the scatter formation phase, which together establish an efficient multi-triangular ring topology. During the leader election phase, the nodes exchange messages to gather node IDs, update tables, and elect a coordinator through a competitive process. Simultaneously, the scatternet formation phase determines the rings based on the coordinator information and predefined rules. The master nodes with the highest neighbor counts initiate unicast connections by following a role table to form piconets within the scatternet. The intra- and inter-bridge nodes then interconnect the piconets, creating a multi-triangular ring scatternet. MTRSRP also includes a self-routing addressing scheme that optimizes the packet transmission efficiency. The key contributions of MTRSRP are summarized as follows:

(1) Decentralized Control

MTRSRP employs decentralized topology control, enabling nodes to autonomously elect a coordinator and form a multi-triangular ring structure.

(2) Competitive Leader Election

MTRSRP uses a competitive leader election process to ensure robust and efficient coordinator selection based on neighbor node information.

(3) Multi-Triangular Ring Scatternet

MTRSRP dynamically determines the number of rings and selects the master nodes with the highest neighbor counts during scattering, facilitating a cohesive multi-triangular ring structure.

(4) Dual Bridge Connections

The protocol utilizes dual intra-bridge and inter-bridge connections for seamless communication within and between piconets, thereby enhancing network connectivity and coverage.

(5) Self-Routing with Shortest path

MTRSRP integrates self-routing functionality within the triangular ring architecture, optimizes packet transmission paths, and reduces overhead by leveraging the established master/slave relationships during scatternet formation.

Overall, the primary contributions of MTRSRP include enhancing the transmission rate, optimizing routing paths to reduce end-to-end delay, and improving network efficiency in multi-ring scatternets compared with other schemes, such as cluster-based on-demand routing protocol (CORP) [12] and Bluetooth low-energy mesh (BLEmesh) [13], through decentralized control and self-routing.

The remainder of this paper is organized as follows. Section 2 provides an overview of the related research on topology construction and routing methods specific to BLE networks. Section 3 explores the design rationale, discusses piconet and scatternet designs, and defines the objectives of the MTRSRP network. Section 4 presents the MTRSRP topology control strategy developed to establish effective scatternet connections and customized self-routing schemes to enhance the efficiency of data packet transmission within such network configurations. Section 5 details the construction of the MTRSRP topology and evaluates the network routing performance using computer simulations. Finally, Section 6 presents the conclusions drawn from this study and outlines the contributions of the proposed protocol.

## 2. Related Work

A notable surge in interest in BLE technology has been observed in recent years. The conventional star topology design has demonstrated limitations regarding network coverage and diversity of available end-to-end paths. Consequently, alternative technologies have been extensively explored to overcome these constraints, particularly through the use of mesh-based approaches for IoT applications [14]. Two primary strategies have emerged in BLE networks to address coverage limitations. The first strategy focuses on expanding the radio range within an existing star-based topology by adjusting the BLE bandwidth in the physical layer. Conversely, the second strategy involves implementing a BLEmesh network, which introduces complexities in the mesh topology configuration but effectively overcomes the coverage and path diversity limitations inherent in a star topology.

A novel on-demand multi-hop routing protocol was introduced in [10] and was specifically tailored for BLE in wireless sensor networks (WSNs). Initially, the network nodes alternate between advertisers and scanners to identify nearby nodes. Subsequently, the source nodes send route request messages to their designated master nodes to locate the destination node and determine the shortest path for packet transmission. This methodology is suitable for implementing the BLE routing protocol in hardware, and its effectiveness was verified through a performance evaluation in a scatternet comprising ten BLE nodes.

BLE technology presents an intriguing opportunity to develop cost-effective industrial WSNs, offering advantages such as low energy consumption and high adaptability. A previous study [15] outlined a configuration approach that ensured controlled message latencies within star topologies. This method involves implementing a mesh-based topology over BLE to maintain limited latencies during packet transmission. Furthermore, to facilitate real-time communication, the integration of a multi-hop real-time BLE (MRT-BLE) protocol within BLE was proposed, prioritizing bounded packet delays, particularly for mesh networks [16]. This paper presented the experimental findings from a realistic test environment and analyzed the worst-case end-to-end packet delay scenarios for MRT-BLE.

Another study [17] proposed a method for addressing dynamic address allocation and network topology mapping in BLE environments. This approach integrated an automatic configuration protocol for defining and managing IDs along with a proactive source routing algorithm for route discovery, maintenance, and mapping within the spanning tree setup. To assess the practicality of energy constraints in BLE, the performance of a battery-limited Bluetooth mesh protocol was evaluated using real hardware to establish models for current consumption, lifespan, and energy expenditure per bit [18]. Furthermore, this study investigated the applicability and limitations of the Bluetooth mesh as an IoT solution considering the power supply leveraging the Bluetooth mesh architecture.

A previous study [12] introduced a CORP topology to facilitate multi-hop communication within BLE networks. Using cluster-based flooding, the protocol effectively manages packet flooding during route discovery, reducing energy consumption compared with traditional flooding methods. This is particularly advantageous because conventional flooding schemes may lead to significant route discovery overheads because packets are forwarded by any node within the mesh connections. Furthermore, a dual-ring tree approach [7] was proposed for BLE networks by incorporating predefined parameters to establish the desired dual-ring tree topology based on single- and multi-hop scenarios. Distributed and localized algorithms have been employed in dense areas to handle multi- and single-hop communication. The resulting dual-ring subnet formed the core network, which was then expanded by a tree-based subnet to cover the less-populated regions. Consequently, this approach enhanced fault tolerance in link connections and improved throughput performance compared to BlueHRT [6].

BLEmesh technology [13] has garnered significant attention from academia and industry and has emerged as a standard solution for BLE multi-hop networks. The managed flooding approach implemented in the Bluetooth mesh effectively reduces the number of route discovery packets, ensuring acceptable end-to-end packet latency. In [19], an evaluation of the Bluetooth mesh’s quality of service and scalability performance was conducted, offering general guidelines and configuration recommendations. By analyzing the timing randomization of the scanning and advertising processes, this study explored the relationship between the reliability, delay, and scalability parameters within Bluetooth mesh protocols. However, scalability remained challenging because of the broadcast storm problem associated with flooding protocols, particularly in densely deployed BLEmesh networks that impose reliability restrictions.

In [20], a new nonuniform power formation algorithm (NUPFA) was proposed to establish a mesh topology to create energy-efficient BLE networks. This method incorporates two levels of power control and introduces dual links between piconets to enhance the energy efficiency and achieve the desired number of piconets through a distributed formation approach. Furthermore, the power consumption required for packet routing within the resulting mesh topology was determined. By leveraging non-uniform power levels and dual links, NUPFA demonstrated reduced packet power consumption and end-to-end packet delay compared to CBM and Bluetooth meshes.

The research presented in [21] introduced a novel approach called the multiple mesh ring (MMR) topology, which was combined with hybrid routing. By employing a predefined mesh ring structure, the MMR methodology achieved balanced connectivity between piconets and scatternets in load distribution. This study also proposed a self-routing protocol to operate across different levels of the mesh ring configuration to minimize routing overhead. To further optimize load-balancing networks using the MMR approach, distributed topology control with a hierarchical self-routing strategy (DTC-HSR) was introduced in [22]. The DTC-HSR protocol integrates the leader selection and topology construction processes to establish a mesh ring topology, thereby ensuring improved performance and operational efficiency. Nonetheless, the routing mechanism within the self-routing protocol can be further refined by incorporating shortest-path optimization techniques.

While our work focuses on BLE networks, we acknowledge that label-based routing has been extensively explored in other domains. For example, PortLand [23] adopted a centralized label assignment scheme to enable scalable Layer 2 routing in data center networks, whereas LESS [24] introduced a distributed label switching mechanism to enhance fault tolerance and reliability. More recently, label-based routing using topology-aware MAC addresses has been proposed for low-power wireless networks, such as the 2021 study [25], which outperformed RPL by embedding routing information directly into MAC address structures. Although these techniques have inspired aspects of our design, MTRSRP differentiates itself by offering a lightweight, fully decentralized labeling mechanism specifically tailored for hierarchical BLE scatternets, which are constrained by energy and hardware limitations.

This paper presents an integrated framework that combines a decentralized multi-triangular ring topology with a decoding-based self-routing protocol to enhance BLE network performance. The proposed design optimizes the number of rings and utilizes a dual-bridge architecture to enable efficient, shortest-path routing. By leveraging the balanced connectivity inherent in piconets and scatternets, the multi-triangular ring topology effectively manages routing traffic, improves load balancing, and achieves superior performance compared to conventional BLE topologies.

In comparison with recent protocols, DTC-HSR adopts a cluster-based hybrid approach involving semi-centralized topology control and layered routing, while NUPFA focuses on probabilistic forwarding and non-uniform power control within unstructured networks. MTRSRP, on the other hand, emphasizes deterministic self-routing through binary label decoding supported by a structured and decentralized topology. This distinction highlights the novelty and advantages of MTRSRP over existing state-of-the-art methods. A summary of these protocols, including MTRSRP, is provided in Table 1.

## 3. Motivation

Traditional Bluetooth piconet structures typically include star, bus, ring, and mesh configurations, with the ring-based configuration demonstrating superior throughput performance, especially under high-traffic loads, compared with the star or bus configurations. Therefore, a ring-based configuration is preferable considering data transmission performance. Each ring subnet can also be interconnected via bridges to construct a large-scale multi-ring scatter network suitable for various applications.

This study addresses the issue of topology construction in smart home network applications. Portable devices, smart meters, household appliances, and plugged-in electronic devices can autonomously form networks using BLE technology. Upon activation, each device is operated by a user and anticipates receiving a “network connection” message while also being capable of exchanging collected information with other devices. By implementing efficient topology construction protocols, an application can achieve advanced levels of connectivity within the home network.

To satisfy the constraints of forming a scatternet, ideal protocol characteristics should facilitate the construction of topological structures under various specific application standards. For example, the nodes can play different roles in different application scenarios. Similarly, a device may impose more restrictions based on its role. To meet traffic demands, standards must also be designed to form participating nodes with scatternet formations that are largely dependent on specific applications. To develop a load-balancing protocol with a balanced connectivity configuration suitable for both piconets and scatternets, this study proposes the following constraints to construct multiple networks, resulting in a topology structure that is easier to control:

(R1) According to the Bluetooth Core Specification, each master node can support up to seven active slave nodes due to the 3-bit address space (AM_ADDR). This constraint ensures stable scheduling and connection management under time-division duplexing (TDD).
(R2) Each piconet is connected to others through slave/slave (S/S) bridge nodes. This supports stable scatternet construction without routing overhead, especially during topological changes, and improves resilience through decentralized ring-based connectivity.
(R3) Each bridge node is limited to connecting two piconets to reduce time-division switching complexity, avoid communication overload, and minimize energy consumption on resource-constrained BLE devices.
(R4) Limiting the connections between two piconets to one or two bridges helps define the minimum number of required piconets and ensures deterministic termination of the formation protocol. It also avoids routing loops and supports modular ring construction.

## 4. The Proposed MTRSRP

### 4.1. System Architecture

The MTRSRP system architecture utilizes multi-tiered rings to organize nodes into structured layers, thereby enhancing routing and connectivity. The key components are:

- Piconets: Fundamental network units, each comprising one master node and several slave nodes;
- Rings: Higher-level structures comprising interconnected piconets managed by intra- and inter-ring bridges;
- Coordinator: A master node with additional responsibilities, such as collecting network data and coordinating the formation of the scatternet.

The MTRSRP method includes two primary phases to establish the network topology: leader selection and scatternet formation.

The leader selection phase involves selecting the coordinator node through a node discovery process and establishing the initial piconet structures, which are detailed as follows.

(1) Node Discovery: Nodes alternate between advertiser and scanner roles to discover neighbors and exchange IDs and neighbor counts. Advertisers broadcast their IDs on designated channels, and scanners collect the information. This process continues until the nodes fully understand their immediate neighbors.
(2) Coordinator Election: Nodes compete to become coordinators based on the collected neighbor information. The node with the highest neighbor count is elected as the coordinator. The coordinator then gathers timing information, node addresses, and neighbor counts from all the nodes.

In contrast, the scatternet formation phase utilizes the coordinator’s information to form multi-tiered ring structures that efficiently connect the piconets. Specifically, the coordinator uses the collected data to organize the nodes into interconnected rings. Each ring comprises multiple piconets connected by intra-ring bridges, whereas inter-ring bridges link different rings, creating a multitiered structure.

Additionally, the self-routing protocol of MTRSRP uses coding-addressing techniques to optimize packet transmission paths. This protocol ensures that packets follow the shortest path within triangular rings and between rings, thereby reducing communication latency and enhancing network efficiency.

In conclusion, the architecture and phases of the MTRSRP method were designed to optimize the formation and routing of BLE networks. The protocol aims to improve routing efficiency, reduce latency, and enhance network stability by organizing nodes into structured multi-tiered rings and using a well-defined leader selection and scatternet formation process.

### 4.2. Multi-Triangular Ring Formation

In this scenario, all bridges are of the S/S type and are restricted to connecting only two piconets. They are classified into two roles based on their positions: the intra-ring bridge (B_intra), which sequentially connects piconets within the same ring, and the inter-ring bridge (B_inter), which links piconets from different rings to form a network comprising multiple interconnected rings. These rings are numbered from the innermost layer (layer 0) to the outermost layer (layer *Nr*).

**Figure 1:**
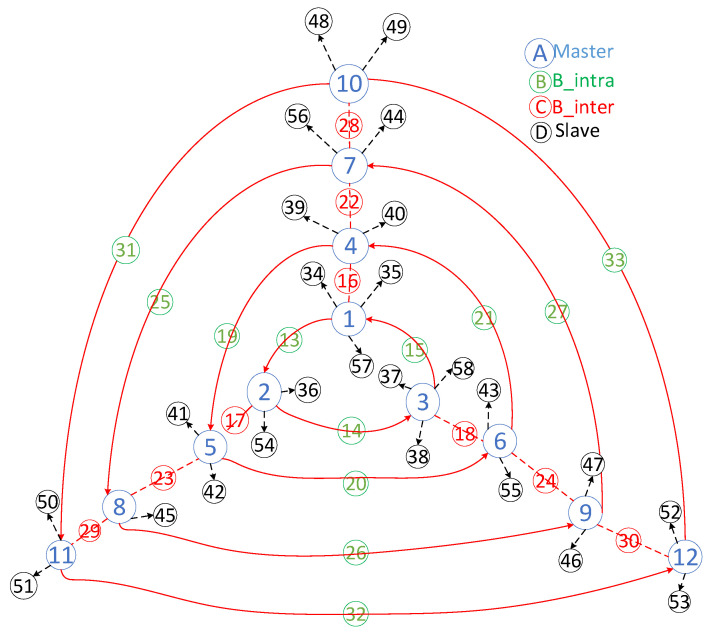
An example of four interconnected triangular ring networks.

Figure 1 provides an interconnected example of four triangular ring networks, demonstrating the connection layout of the multi-ring networks and the functions of each component. In this illustration, the blue nodes denote the master nodes, green nodes signify the intra-ring bridge nodes (B_intra), red nodes represent the inter-ring bridge nodes (B_inter), and black nodes indicate the slave nodes. According to R7, each ring typically links three piconets, resulting in core components of each ring comprising three master nodes and three B_intra nodes.

**Figure 2:**
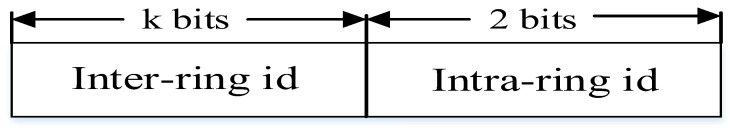
The packet format for piconet and scatternet addressing.

In addition, we designed a binary encoding mechanism for each piconet (based on the master unit). Encoding was divided into two parts. The first part encodes the piconets on different rings, as described previously. Starting from layer 0 and proceeding with binary coding from the innermost to the outermost ring, a k-bit binary code can encode 2*^k^* rings. In principle, *Nr* should be less than or equal to 2*^k^*. The code between the rings is called the inter-ring ID and is placed at the beginning of the first part, forming a complete encoding mechanism. The second part encodes piconets at different positions within each ring. This part requires only two fixed bits encoded in a counterclockwise direction, referred to as the intra-ring ID, specifically 01, 11, and 10. Masters with the same intra-ring ID on different rings are connected through an inter-ring bridge node (B_inter). Figure 2 shows the address of the encoded packet format for the piconet and scatternet.

Once the design framework and coding rules are established, and with knowledge of the total number of network nodes N, the calculation of a certain number of *Nr* is performed based on the constraints outlined in R1–R4. This calculation enables the formation of a multi-ring network, which is then used to implement a self-routing protocol for data packet transmission. Subsequently, the bridge node design for the multi-ring network was categorized into three cases:

Case 1: Only one B_inter and one B_intra bridge exist between two piconets.

Case 2: One B_intra bridge connects two piconets within a ring, whereas two B_inter bridges link piconets across the ring.

Case 3: Two B_intra bridges interconnect two piconets within a ring, and one B_inter bridge connects the piconets across the ring. However, designing two B_intra and two B_inter bridges is not feasible, as it would result in the number of nodes within some piconets exceeding seven, which violates constraint R1.

*CASE 1: ONLY ONE B_INTER AND ONE B_INTRA BRIDGE EXISTS BETWEEN TWO PICONETS*.

Based on the constraints outlined in R1-R4, a relationship between the number of nodes (N) and number of rings (*Nr*) can be established, as shown in Table 2. This table outlines the minimum and maximum number of nodes required to vary the ring numbers, thereby facilitating the derivation of Equation (1). Conversely, if the number of network nodes (N) can be obtained, Equation (1) can be used to calculate the potential number of rings (*Nr*) formed.

(1) $$9\times N_{r}-3\leq N\leq 18\times N_{r}+3$$

The derivation process of Equation (1) can be categorized into scenarios with minimum and maximum numbers of nodes, as explained below.

Each basic ring member has six nodes comprising three masters and three intra-ring bridges. To achieve the minimum number of basic members, three B_inter bridges must be added between the rings. In addition, each piconet can accommodate additional slave members. For the innermost and outermost rings, where the masters are connected to two B_intra and one B_inter, respectively, considering that each piconet can connect up to seven nodes, these rings can accommodate four slave members each, totaling eight slaves. For the remaining rings, excluding the innermost and outermost rings, the masters were connected to two B_intra and two B_inter bridges, allowing each piconet to include up to three slave members. The total number of slaves for these rings was $(N_{r-2})\times 3\times 3$. Adding these numbers to the minimum number of basic members yields the maximum node capacity, which is the sum of these numbers: $18\times N_{r}+3$.

Based on Table 2 and Formula (1), the networks with the same number of nodes N can exhibit varying numbers of rings *Nr*, which could be m − 1, m, or m + 1. For example, if N = 33, the network can potentially produce two, three, or four rings. In such a scenario, selecting a network configuration with the minimum number of rings is preferable. This decision was guided by the principle that a higher number of rings implies more piconets, leading to increased operational costs. Therefore, the least number of rings were selected as the most efficient configuration for MTRSRP.

*CASE 2: ONE B_INTRA BRIDGE CONNECTS TWO PICONETS WITHIN A RING, WHEREAS TWO B_INTER BRIDGES CONNECT PICONETS ACROSS THE RINGS*.

Following constraints R1–R4, Table 3 outlines the relationship between the number of nodes and number of rings for Case 2. Additionally, using Table 3, Equation (2) can be deduced.

**Figure 3:**
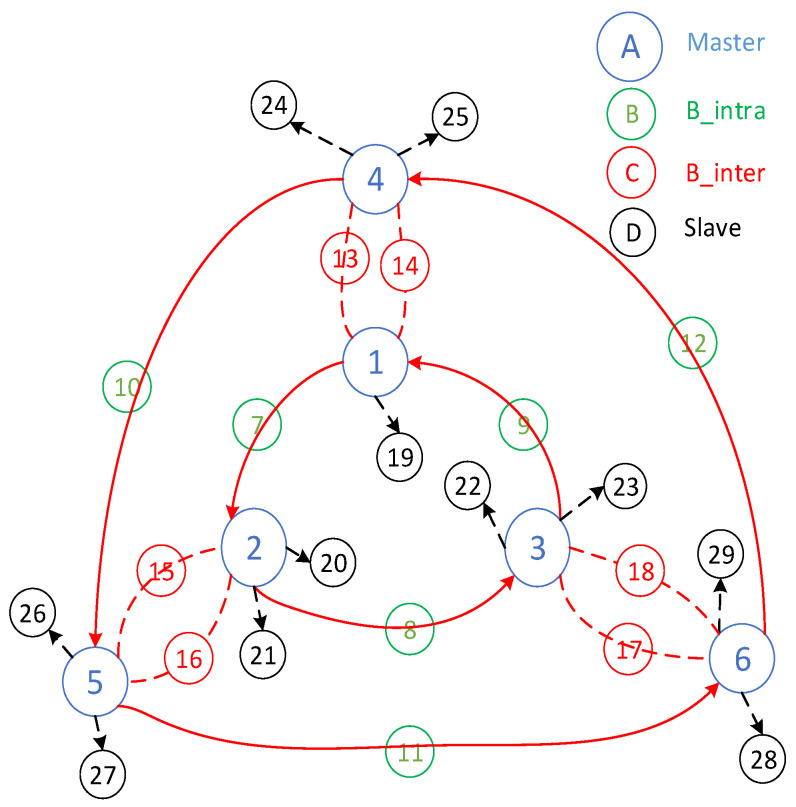
An example of two interconnected triangular rings with dual inter-bridges for case 2 networks.

The derivation of Equation (2) is as follows. The initial ring contains six basic members, including three masters and three intra-ring bridges. With each additional ring, the number of basic members increases by 12, comprising three masters, three B_intra bridges, and six B_inter bridges between rings. Thus, the minimum lower limit of the basic members was $12\times N_{r}-6$. In addition, each piconet can include additional slave members. For the masters on the innermost and outermost rings already connected to two B_intra and two B_inter bridges, each piconet can add up to three slave members, totaling 3×3×3 slave members. Except for the innermost and outermost rings, the piconets on the other rings are connected to two B_intra and four B_inter bridges, allowing each piconet to add at most one slave member, totaling $(N_{r}-2)\times 1\times 3$ slaves. Adding these to the minimum basic members yielded the maximum limit of the nodes that could be accommodated, which was $16\times N_{r}+6$ nodes. Figure 3 illustrates an example of the two interconnected triangular ring networks with dual inter-bridges in Case 2.

(2) $$12\times N_{r}-6\leq N\leq 15\times N_{r}+6$$

*CASE 3: TWO B_INTRA BRIDGES INTERCONNECT TWO PICONETS WITHIN A RING, AND ONLY ONE B_INTER BRIDGE CONNECTS PICONETS ACROSS THE RINGS*.

Based on the above constraints, the data in Table 3 can be derived, which outline the relationship between the number of nodes and number of rings. In addition, Equation (3) can be derived from the data presented in Table 4. The relationship between the number of nodes (*N*) and number of rings ($N_{r}$) for Case 3 is:

(3) $$12\times N_{r}-3\leq N\leq 15\times N_{r}+3$$

**Figure 4:**
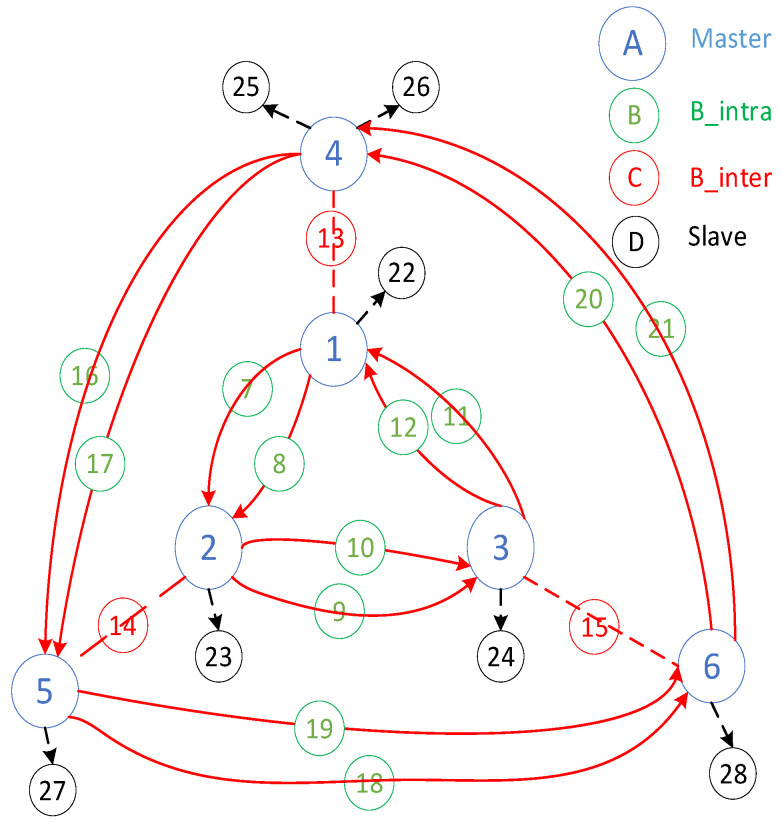
An example of two interconnected triangular rings with dual inter-bridges for Case 3 networks.

The derivation process of Equation (3) can be derived as follows. Each basic member in a ring comprises nine nodes with three masters, six B_intra bridges, and three B_inter bridges between the rings. This provides the minimum basic membership of $12\times N_{r}-3$. Each piconet can accommodate additional slave members. The masters in the innermost and outermost rings are already connected to four B_intra and one B_inter bridge, allowing each piconet to add two slave members, totaling 2×3×3. For rings other than the innermost and outermost ones, the piconets are connected to four B_intra and two B_inter bridges, allowing each piconet to add at most one more slave member, totaling $\left(N_{r}-2\right)\times 1\times 3$ slaves. Adding these to the minimum basic members yields the maximum limit of the nodes that can be accommodated, which is $5\times N_{r}$ + 3 nodes. An example of two interconnected triangular ring networks with dual intra-bridges in Case 3 is shown in Figure 4.

The operational scenario discussed in this study assumes that all nodes are within the transmission range. In such an environment, node mobility has a relatively minor impact on the network architecture because significant issues are avoided if the nodes remain within the communication range. To simplify this problem, the next step involves determining a method for establishing the desired network architecture. The process of creating a multi-ring interconnected network is divided into two main phases: (1) leader selection, which focuses on choosing a coordinator and providing them with information such as timing, addresses, and other relevant details about all nodes within the network, and (2) a scatternet formation phase, which utilizes the information provided by the coordinator, along with the three identified cases, to establish connections between nodes and facilitate packet transmission.

(1) The leader election phase:

In the initial stage of forming a Bluetooth network, establishing point-to-point connections requires neighbor node discovery and information exchange. BLE devices accomplish this by exchanging broadcast messages to collect the IDs and numbers of neighboring nodes. When nodes receive broadcast messages from their neighbors, they update their neighbor tables according to the number of neighbors. This study employs this process to select a coordinator using a competitive method and achieve information aggregation. This phase is divided into node discovery (Step 1) and coordinator selection (Step 2).

Step 1: Initially, each node randomly assumes the role of the advertiser or scanner and then switches roles after a specific duration, called the discovery timer T1, with a fixed operational timer T2, meeting the maximum advertisement interval specified in the BLE 4.1 standard. The procedure involves advertisers broadcasting messages on channels 37, 38, and 39, which contain node IDs and neighbor counts. The scanning nodes receive these advertisement messages on channels 37, 38, and 39 and update their neighbor counts and tables accordingly. These neighbor counts and tables are then used in the next broadcast round. Initially, the neighbor count was set to zero. Algorithm 1 lists the pseudocode for the node discovery process. After exchanging information during the discovery cycle, any two nodes designate the node with the highest neighbor count as the master and the other as the slave. The master then collects information regarding the other nodes owned by the slave.
**Algorithm 1.** Pseudocode of the node discovery process.**Input:** *N* nodes are randomly deployed in a given area **Output:** Several masters are elected to own the neighbors’ information **Init:** Start discovery timer *T*1**while** (*T*1 *>* 0)  Nodes Start timer *T*2 and randomly act as advertisers or scanners  **if** (*T*2 *>* 0)    **if** (Random_op = advertiser)      Advertising its own ID and the number of neighbors    **else**
      Scanning channels 37, 38, and 39 to update neighbor list    **end if**  **else**
    Switch roles and repeat the above advertising and scanning process    **if** any two nodes exchange information with each other      Compare their neighbor lists        **if** the node has the most neighbors, it becomes a master and resets timer *T*2        **else** the node becomes a slave        **end if**    **end if**  **end if** **end while**

Step 2: This step involves temporarily locking slaves to prevent them from establishing connection links and exchanging information with other nodes. Only the masters can re-enter the node discovery process from Step 1, competing for the master role based on higher neighbor counts and gathering information about the other nodes owned by the other master. Steps 1 and 2 are repeated until the final master, known as the coordinator, is determined at time T. The coordinator then possesses critical information about all other nodes in the network, including the timing, addresses, and number of nodes N. Algorithm 2 shows the pseudocode for the super-master selection process.
**Algorithm 2**. Pseudocode for determining a coordinator.**while** (the final winning master is not determined and *T*_2_ < *T*)  **If** *p* > 0.5 in each master,    the master is assigned to the scan mode  **else**    the master is assigned to the advertise mode  **end if**  **if** any two masters exchange information with each other    Compare their neighbor list    **if** the number of neighbors is equal      The winner is the node with the smaller Bluetooth ID, and becomes as a master    **else** the node with the most neighbors becomes the winning master      The losing node becomes as slave, and will deliver its neighbor list to the winner.      The losing nodes are removed until the final winning master is determined.    **end if**  **end if** **end while**

(2) The scatternet formation phase:

A two-stage selection scheme is proposed in this phase. First, the number of rings (*Nr*) is determined based on the number of nodes N, which is collected by the coordinator, and the relationship between the number of nodes and rings in the three cases. *Nr* is calculated using Equations (1)–(3). Subsequently, up to $3\times N_{r}$ master nodes are selected, each with the highest number of neighbors, including the coordinator itself. Depending on the selected case, the coordinator computes a role table for each master node, records the roles of each node in the piconet, and distributes this table to each master node. This table contains information on B_intra and B_inter and the average distribution of slaves for each master in a piconet. From this table, the master nodes can send unicast connection request messages to the recorded members starting with the device with the smallest ID. This process establishes a piconet for each master and connects them to other masters in intra- and inter-ring scenarios, ultimately forming a scattered network. Algorithm 1 presents the pseudocode for the role allocation and formation process of the multi-ring network. Algorithm 2 presents the pseudocode for determining a coordinator and connecting the piconets to the multiple-ring networks.

### 4.3. Self-Routing Protocol of MTRSRP

With the continuous development of network communication technology and the increasing demand for applications in recent years, the effective design of network architectures to improve packet transmission efficiency and reduce communication latency has become a topic of interest in both academia and industry. In this context, MTRSRP combines multiple triangular ring network structures with coding addressing techniques to achieve optimal self-routing paths and enhance the overall efficiency of network packet transmission.

To explain the operation of this self-routing protocol, the encoding mechanism described above was employed because BLE devices depend on transmitting data through their respective piconet masters. This strategy helped streamline the data forwarding process by directing packets from the source master (SM) to the destination master (DM). In addition, an intermediate master (IM) was introduced to represent masters with identical intra-ring IDs that connect different rings, indicating their linkages via B_inter bridges. The self-routing strategy involves transmitting packets from the SM through B_intra to the corresponding IM on the ring of the DM and then forwarding them to the DM via B_inter. This process relies on the aforementioned coding mechanisms to address each piconet. The encoding tasks can be completed during the coordinator’s master node calculations, and the results are recorded in a role table sent to each master for the piconet and scatternet. The pseudocode of the self-routing strategy is described in Algorithm 3.
**Algorithm 3.** Pseudocode of the binary label assignment procedure.**Procedure**: AssignBinaryLabels(RootNode, MaxRings, K) **Input**: RootNode: coordinator node, MaxRings: maximum number of rings, K: branching factor per node **Output**: LabelMap: mapping from each node to its full binary label (InterID + IntraID) Initialize Queue ← [RootNode] Set LabelMap[RootNode] ← “000000”  // *k* bits InterID + 2 bits IntraID Set RingLevel[RootNode] ← 0 **While** Queue is not empty:   Parent ← Queue.dequeue()   ParentLabel ← LabelMap[Parent]   CurrentRing ← RingLevel[Parent] + 1   **If** CurrentRing ≤ MaxRings then      **For** i from 1 to K do         Child ← GenerateChildNode(Parent, i)           **If** Child ≠ null then               InterID ← Binary(CurrentRing, *k*) // fixed *k* bits              IntraID ← Binary(i, 2) // fixed 2 bits                   Label ← InterID + IntraID                  LabelMap[Child] ← Label                  RingLevel[Child] ← CurrentRing                  Queue.enqueue(Child)          **end if**      **end for**   **end if** **end while** **Return** LabelMap

**Figure 5:**
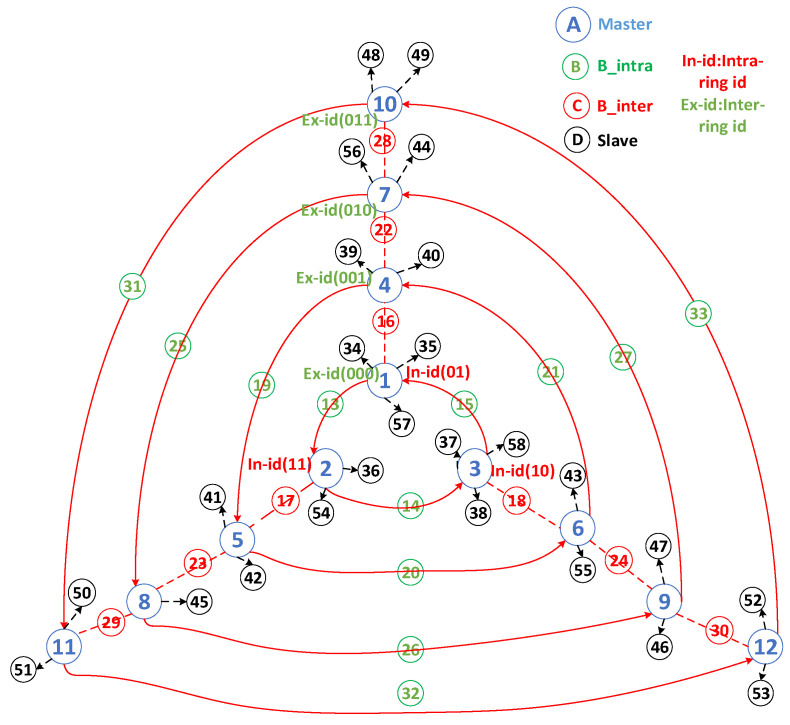
An example of self-routing packet forwarding in MTRSRP.

Figure 5 shows the self-routing method. This study considered the transmission of packets from node 49 (SM) to node 42 (DM) with piconet codes “01101” and “00111,” respectively. The last two digits of their codes indicate that node 42 is positioned counterclockwise from node 49. By utilizing the B_intra connection between these nodes, packets from node 49 can be routed to node 11 (code: 01111), which acts as an IM. Subsequently, the process from the IM to the DM was determined by comparing the first three bits of the codes. As “011” decreased to “001,” the transmission shifted from the outer ring to the inner ring, traversing through layers of B_inter bridges until reaching the preceding master of the destination DM and then reaching DM. Hence, the complete transmission process is as follows: 01101 -> 01111 -> 01011 -> 00111.

Another example is the transmission of packets from an inner to an outer ring. This study considered node 38 (SM) with the piconet code “00010,” aiming to send data to node 49 (DM) with the piconet code “01101,” where the intermediate node (IM) is “00001.” The last two digits of their codes indicate that node 49 is positioned counterclockwise from node 38. By utilizing the B_intra connection between these nodes, the packet can be routed to “00001” (IM). Comparing the initial bits of the codes, as “000” increased to “011,” the transmission transitioned from the inner ring to the outer ring. Therefore, the transmission path was as follows: 00010 -> 00001 -> 00101 -> 01001 -> 01101.

In designing MTRSRP, considerations were primarily focused on the connections within triangular rings and between rings. For connections within triangular rings, the design principle of the shortest path was employed by utilizing coding addressing techniques to optimize connection paths. This allows the packets to be transmitted within triangular rings via the shortest path, reducing communication latency and improving packet transmission efficiency. By contrast, a straight-line transmission approach was adopted for connections between rings, which was also based on the shortest path principle, to facilitate efficient packet transmission between rings. The combination of these approaches established self-routing paths for the entire multi-triangular ring network, which aligned with the paths of the minimum spanning trees [23], ensuring communication efficiency and performance of the entire network. Thus, the self-routing path protocol designed by MTRSRP, which utilizes the shortest paths for packet routing in conjunction with coding addressing techniques, significantly enhances the efficiency and performance of network communication, opening up new possibilities and values in the field of network communication technology development and applications.

## 5. Network Performance

### 5.1. Simulation Setup

This simulation involved the development of a discrete event simulator within a MATLAB R2020a environment to assess the performance of multi-ring networks under various conditions. The simulator was meticulously configured with specific parameters: a simulation scenario involving 45 randomly distributed nodes to evaluate the performance disparity between three multi-ring network design cases and the CORP [12] and BLEmesh [13] schemes. From the perspective of network architecture, the multi-ring network design adopts a hierarchical mesh topology that combines the elements of the mesh and ring topologies. In contrast, the CORP design primarily relies on a mesh topology constructed in a decentralized manner, whereas BLEmesh constructs the mesh topology in a fully distributed manner. In this study, the CORP and BLEmesh methods were selected as benchmarks for a comparative analysis.

During the simulation process, each node uses a fixed transmission range of 10 m and a time-division duplex scheduling mechanism in both the piconets and scatternet considering the frequency-hopping channels between different piconets. This mechanism was employed to avoid packet collisions and optimize the data packet transmission process. The packet generation at each node followed a Poisson process model, with the packet generation rate representing the number of new packets generated per second. Packet generation follows a Poisson distribution with rates ranging from 1 to 10 packets per second to emulate stochastic traffic conditions. To manage these packets, each node is equipped with a first-in-first-out (FIFO) queue with a capacity of 400 data packets. The system randomly selects source–destination pairs for each routing path for packet transmission. A taildrop mechanism was employed when the FIFO buffer capacity reached its limit. This mechanism involves dropping newly received packets at each node to ensure stable operation of the network.

### 5.2. Network Performance of MTRSRP

**Figure 6:**
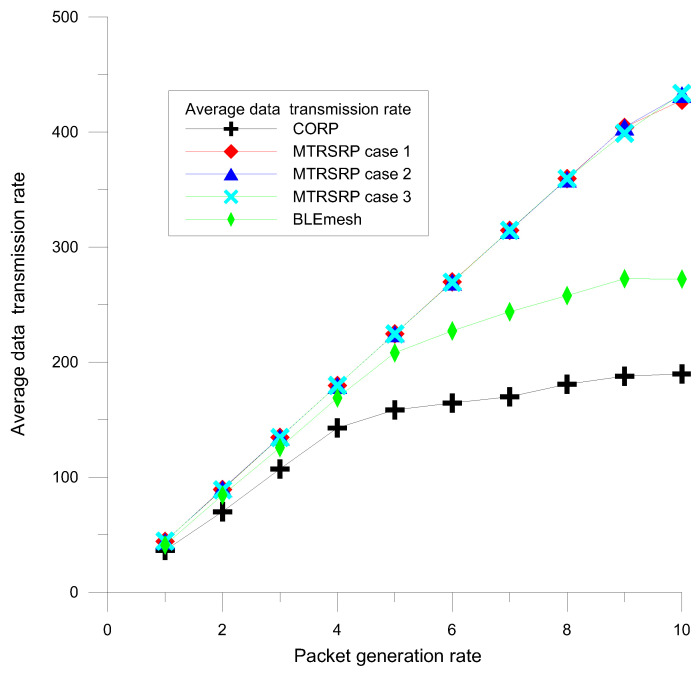
Average data transmission rate performance of MTRSRP, CORP, and BLEmesh.

To evaluate the network capacity of the three bridging methods in MTRSRP, CORP, and BLEmesh, we measured the data transmission rates to assess the transmission performance. The average data transmission rate was defined as the average number of successfully received data packets per second. We simulated the network data rates for Cases 1, 2, and 3 of the MTRSRP network, CORP, and BLEmesh, and the results are presented in Figure 6. The simulation results showed that the network data rate performance improved as the packet generation rate increased. In the MTRSRP network scheme, we observed that increasing the number of grid links in the inter-ring scatter network led to better data transmission rate performance. Compared to CORP and BLEmesh, the multi-ring network demonstrated superior network data rate performance. This is attributed to the multi-path and self-routing mechanisms in the multi-ring network, which make data packet transmission more efficient than the mesh structure of the CORP and on-demand routing methods, thus effectively increasing the overall network capacity. At peak data rates, the multi-ring network with two inter-ring bridges achieves more than double the data rate of the CORP scheme and 1.6 times the data rate of the BLEmesh scheme. This significant improvement is because the multi-ring network establishes more bridged links within the rings, especially in high-traffic areas, which enables higher data transmission efficiency.

**Figure 7:**
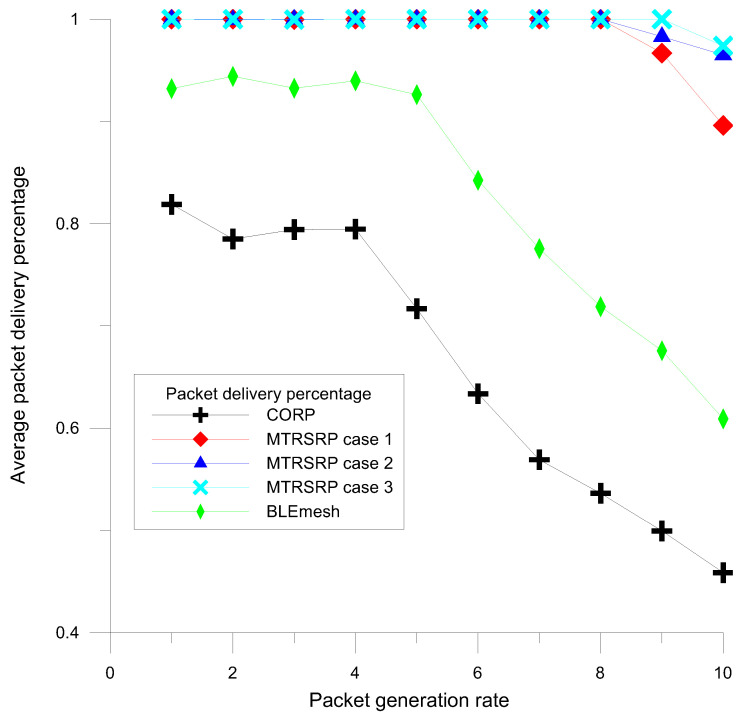
Average packet delivery percentage performance of MTRSRP, CORP, and BLEmesh.

A detailed evaluation of the data reception performance for three different configurations, namely MTRSRP, CORP, and BLEmesh, was conducted. The packet delivery percentage (PDP) is defined as the percentage of successfully received data packets in the network compared to all packets generated by nodes. As shown in Figure 7, MTRSRP demonstrates superior PDR performance compared to those of CORP and BLEmesh as the packet generation rate increases. To comprehensively assess the performance of MTRSRP, this study examined three different configurations under varying numbers of network bridges. The results indicate that in the multi-ring network, an increase in the number of bridges and configurations in Case 3 contributed to an improved data reception performance. Specifically, when the packet generation rate was below 8, MTRSRP achieved approximately 100% PDP, indicating its high routing performance efficiency. This superior performance is attributed to the multi-ring network’s design involving multiple bridging paths and a self-routing protocol, which significantly enhances the routing efficiency of the network and achieves a higher success rate in packet reception.

**Figure 8:**
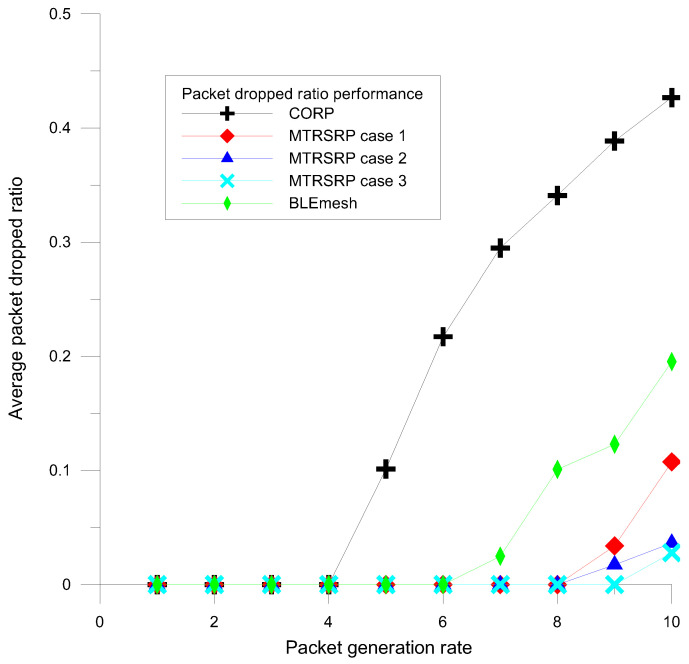
Average packet dropped ratio performance of MTRSRP, CORP, and BLEmesh.

To further explore the load-balancing capabilities of multi-ring networks, this study utilized the packet dropped ratio (PDR) as a key performance metric. The PDR is defined as the ratio of dropped packets to the total generated packets in each network topology. Notably, when the FIFO buffers overflow, the system discards packets, including both currently received and newly generated packets. Figure 8 illustrates a comparison of the PDR performance between the three configurations of the MTRSRP strategy and the CORP and BLEmesh schemes under high-traffic congestion conditions.

The results revealed that the PDR of the MTRSRP was significantly lower than those of the CORP and BLEmesh schemes. This is primarily attributed to the uniformity in the node and link connectivity design in the MTRSRP, which allows for a more balanced distribution of network traffic and reduces the PDR. Furthermore, the slope of the PDR performance in MTRSRP was smoother than those in CORP and BLEmesh. This further demonstrates the advantages of multi-ring networks for load balancing. The multi-ring network effectively mitigates traffic congestion issues that may arise in CORP and BLEmesh because of the random distribution of links in micro-networks through multiple paths and a uniform distribution design.

**Figure 9:**
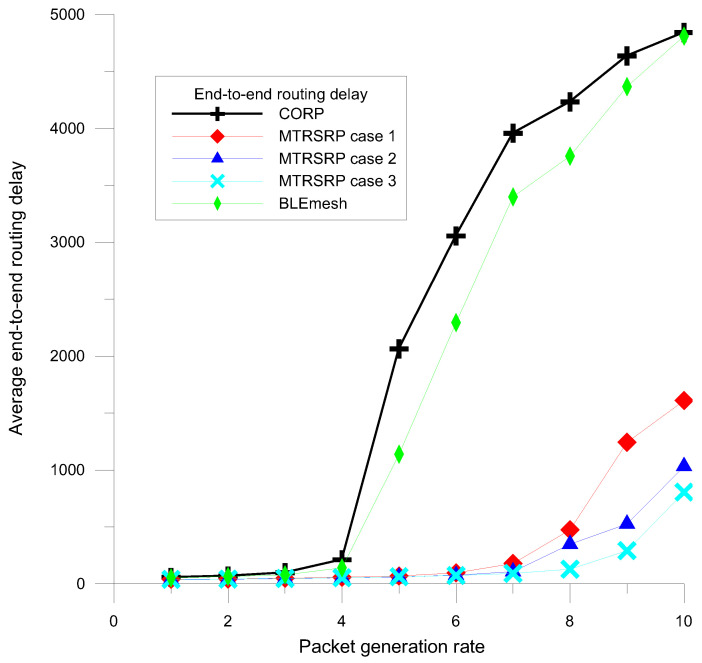
Average routing packet delay of MTRSRP, CORP, and BLEmesh.

Furthermore, to investigate the performance of multi-hop transmission, this study utilized the average end-to-end routing delay as a key performance metric to evaluate the transmission efficiency in the MTRSRP network. Specifically, the routing delay for each data packet is defined as the average time required for data transmission from the initiation of sending the first bit at the source node to the reception of the last bit at the destination node. Figure 9 presents a detailed comparison of the average routing delay performance between the three different topological configurations of MTRSRP, CORP, and BLEmesh.

The results showed that the MTRSRP network significantly outperformed the CORP and BLEmesh networks in this metric. This superiority can be primarily attributed to the bridging of multiple paths and the self-routing mechanism in MTRSRP, which allows shorter path lengths compared to CORP and BLEmesh. The design of multiple bridging paths reduced the average number of transmission hops for self-routing, thereby effectively decreasing routing packet delays. As the packet generation rate increased, the average packet routing delay also increased. However, the multi-ring network exhibited a better mitigation of routing delays under high-traffic loads than CORP and BLEmesh.

When the packet generation rate exceeds 4, as in Figure 9, the routing delay of CORP increases quickly, and its packet drop ratio increases sharply, as shown in Figure 8. Therefore, BLEmesh and CORP must resort to a higher data dropout rate to avoid a sharp increase in packet delay in each node’s data buffer and maintain constant latency. In contrast, a multi-ring network can maintain lower latency while maintaining a lower data dropout rate, ensuring stability and reliability under high-traffic loads.

Although the average delay analysis does not explicitly model the hop count limit defined in BLEmesh specifications (typically 10 hops), the proposed MTRSRP protocol inherently addresses this constraint through its hierarchical triangular ring topology and binary-label-based self-routing mechanism. As shown in Figure 9, MTRSRP achieves a consistently lower average hop count compared to DTC-HSR and NUPFA. In our simulations, the average hop count remains well below the 10-hop threshold under various traffic conditions, ensuring compatibility with BLE protocol limits. Moreover, the binary label structure ensures deterministic and loop-free routing. Each node’s label reflects its unique position in the hierarchical ring topology, and the forwarding logic decodes these labels to compute next-hop decisions in a top-down manner. Since the routing paths are derived from label prefixes and constrained within a defined ring hierarchy, there is no ambiguity or possibility of circular forwarding, thus eliminating routing loops by design.

**Figure 10:**
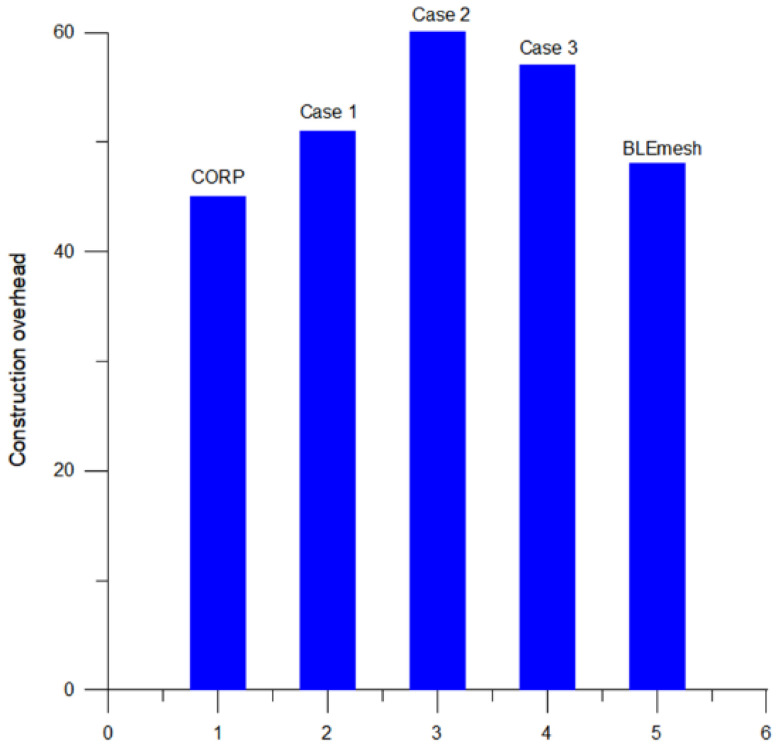
The average number of topology construction overhead of MTRSRP, CORP, and BLEmesh.

Figure 10 illustrates the average number of formation packets generated during the topology construction process for the three configurations: MTRSRP, CORP, and BLEmesh. These construction packets include all the scatternet connection information necessary to establish routing protocols during network topology construction. The total number of construction packets can be viewed as the main communication overhead for establishing the interconnection of the scatternet and designing routing algorithms. As the network scale expands, the number of construction packets increases accordingly.

In MTRSRP networks, the number of packets generated during the topology construction process is relatively high, because it is necessary to establish more connections to form rings and dual-bridge structures. Specifically, the average overhead for the three configurations of the multi-ring network was O(1.24N), which is higher than that of CORP (O(1.0N)) and BLEmesh (O(1.1N)), where N represents the total number of nodes. This indicates that compared to CORP, the MTRSRP network improves network connectivity and reliability by incurring additional communication link costs to form rings and dual bridge links between scatternets.

In summary, the network topology plays a pivotal role in shaping routing efficiency, path optimality, and traffic distribution. The proposed MTRSRP leverages a hierarchical triangular ring structure that offers predictable, loop-free routing paths and evenly distributes load across bridge nodes. This regularized topology reduces average hop count (Figure 9), minimizes packet collisions, and improves packet delivery performance (Figure 7), particularly under high-traffic conditions. Compared to less structured approaches such as BLEmesh and CORP—where routing paths and node connectivity may be irregular—MTRSRP demonstrates more stable and scalable performance.

Furthermore, the evaluation of Cases 1–3 reveals that bridge node configuration significantly impacts routing behavior. Higher bridge density leads to improved path redundancy and traffic balancing, which in turn reduces congestion, lowers latency, and enhances overall delivery success. These findings highlight the importance of topological design and bridge placement in optimizing network performance, reaffirming the advantages of the MTRSRP framework in structured BLE scatternets.

Finally, Table 5 presents a comprehensive comparison of the proposed MTRSRP protocol with two representative BLE-based protocols: CORP [12] and BLEmesh [13]. The comparison covers key aspects such as topology structure, routing approach, stability, and scalability. It highlights how MTRSRP combines a regular multi-triangular ring formation with self-routing based on binary labeling, offering higher throughput, lower packet loss, and better structural scalability under moderate to dense network deployments.

### 5.3. Related Performance Discussions

Although the proposed MTRSRP protocol does not explicitly incorporate an energy consumption model, the performance results in Figure 7 and Figure 9 provide meaningful insights into its energy efficiency. As shown in Figure 7, MTRSRP consistently achieves a higher packet delivery ratio compared to DTC-HSR and NUPFA, even under increasing traffic loads. This improvement implies a significant reduction in retransmissions, which are typically caused by packet losses or acknowledgment failures. In BLE networks, retransmissions are a major source of energy consumption, as they involve repeated transmissions and extended listening periods. By reducing retransmission frequency, MTRSRP indirectly conserves energy across the network.

In addition, Figure 9 reveals that MTRSRP maintains a lower average hop count, indicating more direct and efficient routing paths. Each hop in a BLE network consumes energy for both transmission and reception. Therefore, fewer hops translate directly into lower communication energy expenditure. This efficiency is largely attributed to the protocol’s binary-label-based self-routing mechanism, which ensures deterministic and loop-free forwarding while avoiding redundant or suboptimal paths.

Moreover, the dual-bridge design in MTRSRP enhances energy efficiency by introducing path redundancy and balancing traffic loads across the network. These features help alleviate congestion and minimize packet collisions, which in turn reduce the need for retransmissions and lower end-to-end delay. As a result, the protocol achieves better energy usage without relying on explicit power control mechanisms.

In summary, the high delivery success rate, reduced hop count, and effective traffic distribution enabled by MTRSRP collectively contribute to lower energy consumption. These inherent characteristics make MTRSRP a practical and energy-conscious solution for BLE network deployments.

On the other hand, scalability is another critical concern in BLE network design. To address this, we now provide analytical scalability extrapolations based on Equations (1)–(3) in Section 4.2. The hierarchical structure of MTRSRP is based on interconnected triangular rings, and the total number of nodes N scales approximately linearly with the number of rings *N*r. Through analytical estimation, we find that the average node count can be approximated by N ≈ 13.5⋅Nr. This linear relationship indicates that increasing the number of rings allows the network to scale in a controlled and predictable manner. For example, with eight rings, the network can accommodate more than 100 nodes while maintaining structured routing paths and consistent performance. Such scalability is achieved without significantly increasing routing overhead, as the regular ring-based layout and binary-label routing ensure efficient path planning and minimal forwarding complexity. Therefore, MTRSRP demonstrates strong potential for deployment in large-scale BLE networks where topology regularity and energy efficiency are critical.

## 6. Conclusions and Future Work

This study introduced a novel MTRSRP topology architecture for BLE networks by standardizing a fixed structural pattern and incorporating a convenient encoding mechanism. This mechanism enhanced the formation of the entire network and facilitated data forwarding. This paper details the construction process of the desired MTRSRP formation and introduces self-routing rules. The construction of the MTRSRP topology involves leader selection and scatter formation to establish the desired multi-triangular ring architecture. The self-routing strategy within the multi-ring architecture enables efficient data transmission and eliminates packet looping. The characteristics of the multi-ring interconnected structure mitigate potential root node bottlenecks and resolve problems, such as excessively long network paths caused by a single ring. The experimental results demonstrate that MTRSRP significantly outperforms the CORP and BLEmesh schemes. The MTRSRP network architecture achieves dual-path selection, leading to fewer transmission hops; shorter transmission paths; and improved throughput, end-to-end latency, packet loss rate, and other performance metrics. Despite incurring higher link generation costs than those of CORP and BLEmesh during topology construction, MTRSRP demonstrated superior performance once the network was established. This is attributed to its ability to combine a dual-bridging network structure and self-routing design, highlighting its efficiency and effectiveness in real-world scenarios.

The current design of MTRSRP assumes a relatively static and uniformly distributed deployment environment with sufficient radio coverage and minimal interference. However, real-world BLE applications often involve dynamic topologies, irregular node distributions, and varying wireless channel conditions. To enhance the robustness and adaptability of MTRSRP in such scenarios, future work will focus on three key directions: (1) mobility support: designing a dynamic label re-assignment mechanism and mobility-aware topology adaptation strategy to maintain routing consistency when nodes move; (2) topology flexibility: developing fallback strategies for incomplete triangular layouts, including partial ring formation, redundancy-aware bridge selection, and topology repair algorithms, to ensure connectivity even under sparse or irregular node placement; and (3) link quality adaptation: integrating link quality estimation and adaptive retransmission mechanisms to address the impact of environmental noise and coverage fluctuations on packet delivery and delay performance. These enhancements will broaden the applicability of MTRSRP to more realistic and dynamic BLE network deployments.

## Figures and Tables

**Table 1:** Summary of the main related works, including the proposed MTRSRP.

Refs.	Main Idea	Techniques	Strengths	Limitations
[11]	A layer-based formation and routing algorithm for multihop Bluetooth networks	Layer-based formation, self-routing for multihop networks	Enhance efficiency in multihop networks through joint formation and routing	Complex implementation and potential scalability issues
[12]	A topology configuration and routing protocol for BLE networks	Topology configuration, multihop on-demand routing protocols	Provide efficient topology setup and routing in BLE networks	Limited applicability to other Bluetooth variants
[13]	A standard perspective of BLEmesh networks	Standard discussion, BLEmesh networks	Offer comprehensive insights into BLEmesh standards	Limited to standards discussion, lacks detailed implementation guidance
[20]	Opportunistic forwarding with adaptive power levels	Probabilistic relay selection, non-uniform transmission power control	Energy-aware; adaptable to unstructured deployments	Routing paths are non-deterministic; may suffer from higher delay and routing redundancy
[22]	Hierarchical routing with distributed topology control for BLE scatternets	Clustering, layered backbone formation, semi-centralized coordination	Scalable to moderate-sized networks; reduces route discovery overhead	Relies on synchronized control phases; potential bottlenecks at cluster heads
MTRSRP	A multi-triangular ring topology and a self-routing protocol with dual-paths in BLE networks	Decentralized multi-triangular ring topology formation, self-routing protocols	Optimizes number of rings and the shortest path for packet transmission in BLE networks	Assumes static topology; performance may degrade under high mobility or sparse deployment

**Table 2:** Relationship between the number of nodes N and number of rings $N_{r}$ for Case 1.

Number of Nodes	Number of Rings
6~21	1
15~39	2
24~57	3
33~75	4
……	……

**Table 3:** Relationship between the number of nodes N and number of rings *Nr* for case 2.

Number of Nodes	Number of Rings
6~21	1
18~36	2
30~51	3
42~66	4
54~81	5
……	……

**Table 4:** Relationship between the number of nodes (*N*) and number of rings ($N_{r}$) for Case 3.

Number of Nodes	Number of Rings
9~18	1
21~33	2
33~48	3
45~63	4
57~78	5
……	……

**Table 5:** MTRSRP vs. CORP and BLEmesh.

Feature	MTRSRP	CORP [12]	BLEmesh [13]
Topology structure	Multi-triangular ring	Cluster-based	Flooding-based (unstructured)
Topology formation	Hierarchical competitive leader election	Distributed cluster formation	No explicit topology formation required
Routing method	Self-routing based on binary labeling	Reactive routing based on node roles	Flooding-based message forwarding
Routing stability	High (predefined paths, no reconstruction)	Medium (requires rerouting)	Low (frequent redundant transmissions)
Loop avoidance	Unidirectional labeling ensures no loops	No explicit mechanism; loops possible	None; prone to loops and duplications
Throughput performance	High	Medium	Low to medium (degrades with congestion)
Packet loss rate	Low (multi-path + bridges)	Medium to high (congestion at cluster heads)	High (duplicate/collision prone)
Delay control	Low (shortest-path self-routing)	Medium to high (delay increases under load)	High (lack of congestion control)
Construction overhead	Medium (control packets for ring setup)	Medium to high (distributed control messages)	Low (no structural overhead)
Scalability	Good (regular topology easily extended)	Limited (irregular structure)	High (simple broadcasting mechanism)

## Data Availability

The original contributions presented in this study are included in the article. Further inquiries can be directed to the corresponding author.
